# Serum cardiac troponin T and effective blood flow in stable extracorporeal dialysis patients

**DOI:** 10.1007/s11255-015-1165-z

**Published:** 2015-11-24

**Authors:** Alicja E. Grzegorzewska, Krzysztof Cieszyński, Leszek Niepolski, Andrzej Kaczmarek, Anna Sowińska

**Affiliations:** Chair and Department of Nephrology, Transplantology and Internal Diseases, Poznan University of Medical Sciences, Al. Przybyszewskiego 49, 60-355 Poznan, Poland; Fresenius Nephrocare Dialysis Center, Ostrów Wielkopolski, Poland; B/Braun Avitum Poland, Dialysis Center, Nowy Tomyśl, Poland; Fresenius Nephrocare Dialysis Center, Pleszew, Poland; Chair and Department of Computer Science and Statistics, Poznan University of Medical Sciences, Poznan, Poland

**Keywords:** Cardiac troponin T, Dialysate flow, Effective blood flow, Extracorporeal dialysis, Mortality

## Abstract

**Purpose:**

We examined the association between extracorporeal dialysis (ED)-related effective blood flow (eQB) and serum cardiac troponin T (cTnT) as a possible indicator of silent myocardial damage in stable ED patients.

**Methods:**

In a cross-sectional study, cTnT was determined in 247 ED patients dialyzed using stable eQB and dialysate flow (QD). In a prospective study, 91 patients were switched from low-flux (LF) to high-flux (HF) hemodialysis (HD), and subsequently, the eQB increased, and the QD decreased; 65 patients continued LF-HD with stable eQB and QD. Clinical/laboratory evaluations were performed at 0, 15, 36, and 53 weeks from the start of the study.

**Results:**

In the cross-sectional study, the main cTnT predictors were dialysis vintage, age, eQB, phosphorus, and C-reactive protein. Patients with cTnT levels in the highest quartile were excluded from the analysis, and subjects dialyzed with eQB ≤316 ml/min exhibited lower cTnT levels compared with patients dialyzed with higher eQB (*P* = 0.002). The all-cause and cardiac mortality rates of 154 patients, without changes in ED modality for up to 42 months, were associated with the initial cTnT concentrations but not with the initial eQB. In the prospective study, higher values for eQB and cTnT were observed during HF-HD at weeks 36 (*P* = 0.045) and 53 (*P* = 0.01) of the present study. The initial cTnT, ∆eQB, and ∆albumin influenced the ∆cTnT. The all-cause and cardiac mortality rates were not different between LF and HF groups at 21 months after the prospective study was completed.

**Conclusion:**

In stable ED patients, higher eQB rates and QB/QD values might contribute to silent myocardial injury, particularly in patients with lower cTnT levels, but do not affect the outcome of ED patients.

**Electronic supplementary material:**

The online version of this article (doi:10.1007/s11255-015-1165-z) contains supplementary material, which is available to authorized users.

## Introduction

Cardiac troponins are specific and sensitive markers of ischemic and non-ischemic etiologies of myocardial injury [1]. Elevated concentrations of these markers, particularly cardiac troponin T (cTnT), have been detected in up to 94 % of hemodialysis (HD) subjects [2, 3], reflecting multifactorial contributions [4–11]. Extracorporeal dialysis (ED) might influence serum cardiac troponin concentrations [2, 3, 5, 8, 12–15].

In the present study, we focused on effective extracorporeal blood flow (eQB) rates with respect to serum cTnT concentrations. Previous data and prospective studies concerning this subject are scarce. Artunc et al. [4] conducted a cross-sectional analysis, showing that the plasma cTnT concentration, but not the cardiac troponin I (cTnI) concentration, was positively correlated with the blood pump speed in patients based on the use of high-flux (HF) HD. During HD sessions with a QB of 250–300 ml/min, the observed changes in cTnI were not correlated with the QB rate [13], and a QB of 250–350 ml/min did not influence the predialytic or the interdialytic cTnT concentration [12].

The aim of the present study was to conduct a cross-sectional study to examine the potential correlation between the eQB rate and serum cTnT concentration in patients using different ED modalities and assess whether there is an association between changes in the eQB rate and serum cTnT concentration in a prospective observational study on stable patients treated with HF-HD or low-flux (LF) HD. The results suggest an association between eQB and cTnT; therefore, we also evaluated whether eQB is associated with the mortality rate of ED patients.

## Subjects and methods

### Patients and a study design

In the cross-sectional study, the serum cTnT concentrations were measured in 247 stable ED patients: 54 patients used *on*-*line* HDF and 35 patients were on HF-HD in dialysis center no 1, while the remaining 158 patients were on LF-HD (93 patients were treated in dialysis center no 2, and 65 patients were treated in dialysis center no 3). The demographic data, clinical parameters, including major cardiovascular diseases, and laboratory parameters for the ED patients are shown in Table 1. The dialysis-related parameters for patients treated using different ED modalities differed between groups (Table 2), but remained stable within each group. Among the 247 ED patients, 154 subjects did not show changes in ED modality for up to 42 months since the cross-sectional analysis. The individuals underwent an evaluation of all-cause and cardiac mortality rates with respect to the initial eQB and cTnT.

**Table 1:** Demographic, clinical, and laboratory data of patients treated using different modalities of extracorporeal dialysis

Parameter	HDF	HF-HD	LF-HD	*P* value
	*n* = 54	*n* = 35	*n* = 158	
Male gender (*n*, %)	38 (70.4)	18 (51.4)	82 (51.9)	0.03^a,C^
Age (years)	64.6 ± 12.3	60.6 ± 15.3	64.0 ± 14.4	Ns
Diabetic nephropathy (*n*, %)	20 (37.0)	6 (17.1)	35 (22.2)	0.03^a,C^
Coronary artery disease (*n*, %)	40 (74.1)	11 (31.4)	34 (21.5)	<0.0001^a,C^, <0.0001^b,C^
Myocardial infarction (*n*, %)	27 (50.0)	9 (25.7)	23 (14.6)	<0.0001^a,C^, 0.03^b,C^
Cardiomyopathies (*n*, %)	21 (38.9)	6 (17.1)	44 (27.8)	0.035^b,C^
Valvular disease (*n*, %)	4 (7.4)	3 (8.7)	34 (21.5)	0.02^a,C^
Atrial fibrilation (*n*, %)	3 (5.6)	4 (11.4)	14 (8.7)	Ns
Poor control of hypertension (*n*, %)	7 (13.0)	5 (14.3)	24 (15.8)	Ns
Cerebral stroke (*n*, %)	7 (13.0)	1 (2.9)	10 (6.3)	Ns
BMI (kg/m^2^)	27.1 (18.22–38.16)	23.8 (19.75–47.8)	27.35 (17.75–55.33)	0.01^c,D^
Albumin (g/l)	37 (26–44)	39 (18–44)	42 (25–49)	<0.0001^a,c,D^
cTnT (ng/ml)	0.050 (0.003–0.315)	0.032 (0.008–0.595)	0.047 (0.004–0.410)	0.09^K^
CRP (mg/l)	4.0 (0.4–57.7)	6.39 (0.7–89.5)	7.75 (0.7–241.2)	0.3^K^
β_2_-Microglobulin (mg/dl)	na	2.18 (1.45–2.78)	3.48 (0.09–13.9)	0.07^MW^
Ca, mg/dl	8.6 (5.1–11)	8.4 (6.48–9.52)	8.88 (6.6–11.4)	0.004^c,D^
P (mg/dl)	5.1 (2–10.1)	4.5 (1.6–8)	4.7 (2.4–11.1)	0.07^K^
PTH (ng/l)	245.3 (9.1–2002.2)	230.8 (21.87–1781.9)	299.8 (4–1962)	0.4^K^
ALP (IU/l)	74.5 (42–346)	99.0 (40–322)	95.5 (39–725)	0.02^a,D^
Bicarbonate (mmol/l)	21.7 (13.9–28.8)	22.1 (11.8–30.4)	21.85 (15.1–28.3)	0.7^K^

Results are shown as median and range, mean ± SD, or as numbers with percentage

Statistical tests: C—Chi-square, D—Dunn (post hoc Kruskal–Wallis), MW—Mann–Whitney, K—Kruskal–Wallis

Conversion factors to SI units are as follows: for CRP—9.524, for Ca—0.25, for P—0.323, for ALP—0.0167

*ALP* total alkaline phosphatase, *BMI* body mass index, *CRP* C-reactive protein, *cTnT* cardiac troponin T, *HDF* hemodiafiltration, *HF-HD* high-flux hemodialysis, *LF-HD* low-flux hemodialysis, *na* not available, *PTH* parathyroid hormone

^a^Comparison between HDF and LF-HD

^b^Comparison between HDF and HF-HD

^c^Comparison between HF-HD and LF-HD

A *P* value of <0.05 is considered statistically significant

**Table 2:** Dialysis-related parameters in patients treated using different modalities of extracorporeal dialysis

Parameter	On-line HDF	HF-HD	LF-HD	*P* value
	*n* = 54	*n* = 35	*n* = 158	
Total RRT vintage (years)	2.9 (0.05–22.9)	0.2 (0.02–11.1)	2.6 (0.07–25.9)	<0.0001^b,c,D^
Actual RRT vintage (years)	2.4 (0.05–3.8)	0.2 (0.02–11.1)	2.6 (0.07–24.0)	<0.0001^b,D^ 0.00002^c,D^
Arteriovenous fistula (*n*, %)	50 (92.6)	13 (37.1)	129 (81.6)	<0.0001^b,c,C^
Arm (*n*, %)	3 (6.0)	2 (15.4)	41 (31.8)	0.001^a,C^, 0.01^c,C^
Proximal forearm (*n*, %)	33 (66.0)	4 (30.8)	7 (5.4)	<0.0001^a,C^, < 0.0001^b,C^
Distal forearm (*n*, %)	14 (28.0)	7 (53.8)	81 (62.8)	0.001^a,c,C^
Permanent catheter (*n*, %)	4 (7.4)	22 (62.9)	29 (18.4)	<0.0001^a,b,c,C^
Dialysis session duration (min)	240 (240–300)	240 (180–260)	255 (210–320)	0.05^b,D^, < 0.0001^c,D^
eQB (ml/min)	354.5 (158–420)	317 (162–392)	304 (148–370)	<0.0001^a,D^, 0.003^b,D^
QD (ml/min)	503.5 (280–600)	500 (300–500)	500 (300–800)	<0.05^a,b,D^
QB/QD (%)	69.5 (31.4–89.0)	63.8 (32.4–108.0)	60.8 (29.6–107.3)	<0.0001^a,D^, 0.002^b,D^, 0.03^c,D^
Kt/V_urea_	1.39 (0.92–1.78)	1.25 (0.65–1.99)	1.3 (0.54–1.91)	0.02^a,D^
Body weight (kg)				
Before dialysis	78.9 ± 16.5	71.5 ± 15.8	77.5 ± 17.9	Ns
After dialysis	76.8 ± 16.1	69.9 ± 15.4	75.4 ± 17.5	Ns
Difference	2.1 ± 1.2	1.6 ± 1.1	2.0 ± 1.0	Ns

Results are shown as median and range or as numbers with percentage

Statistical tests: C—Chi-square, D—Dunn (post hoc Kruskal–Wallis)

*eQB* effective blood flow, *HDF* hemodiafiltration, *HF-HD* high-flux hemodialysis, *LF-HD* low-flux hemodialysis, *QD* dialysate flow, *RRT* renal replacement therapy

^a^Comparison between HDF and LF-HD

^b^Comparison between HDF and HF-HD

^c^Comparison between HF-HD and LF-HD

A *P* value of <0.05 is considered statistically significant

LF-HD patients were dialyzed in center no 2 (*n* = 93) and subsequently switched to HF-HD. As a progressive increase in eQB to the values recommended for HF dialyzers by their manufacturer was planned in these patients, when possible, we designed a prospective study concerning the impact of increasing eQB on the serum cTnT concentration. Among 93 patients, 91 subjects provided written informed consent to participate in this prospective observational study. In addition, 65 patients from dialysis center no 3 were enrolled into a similarly designed prospective observational study, but with continuation of LF-HD using stable eQB and dialysate rates (QD) (see details in the “Results” section).

Routine clinical/laboratory evaluations for stable HD patients and serum cTnT and β_2_-microglobulin measurements were performed in both patient groups at 0, 15, 36, and 53 weeks from the start of the prospective study. As patients presenting cardiac episodes with clinical manifestation or other severe instability and individuals who underwent renal transplantation during the present study (Table 3) were excluded, the serum cTnT concentration was analyzed in 44 LF-HD and 58 HF-HD patients. The outcome of these patients was evaluated at 21 months after the prospective study was completed.

**Table 3:** A patient loss during a 53 weeks prospective study

Causes of a patient loss	HF-HD	LF-HD	*P* value
	(*n* = 91)^a^	(*n* = 65)^a^	
Deaths (*n*, %)	15 (16.5)	10 (15.4)	1.0^C^
Cardiovascular mortality (*n*, %)	6 (6.6)	7 (10.8)	0.5^Y^
Non-fatal cardiac episodes (*n*, %)	11 (12.1)	6 (9.2)	0.8^Y^
Instability of non-cardiac origin (*n*, %)	3 (3.3)	4 (6.2)	0.6^Y^
Renal transplantation (*n*, %)	4 (4.4)	1 (1.5)	0.6^Y^
Total	33 (36.3)	21 (32.3)	0.7^C^

Results are shown as numbers with percentage

Statistical tests: C—Chi-square, Y—Chi-square with Yates correction

*HF-HD* high-flux hemodialysis, *LF-HD* low-flux hemodialysis

^a^Initial number of patients

The dietary and pharmacological treatment of all patients was based on a standard of care according to the physician. The results of the clinical/laboratory evaluations performed during the prospective study were used for the appropriate modification of this treatment when necessary.

### ED schedule and modalities

In the cross-sectional study, three bicarbonate dialysis sessions per week were prescribed to all patients, except for two patients, who underwent four sessions per week. Polysulfone hollow-fiber dialyzers F6HPS–F10HPS (Fresenius Medical Care, Bad Hamburg, Germany) or Lo10–Lo23 (B/Braun Xevonta, Melsungen, Germany) were used for the LF-HD patients. Helixone hollow-fiber dialyzers FX_CorDiax_50 or FX_CorDiax_60 (Fresenius Medical Care, Bad Hamburg, Germany) were used for the HF-HD subjects. *On*-*line* HDF was performed using the predilution method and FX_CorDiax_50 or FX_CorDiax_60 dialyzers.

All patients enrolled into the prospective observational study underwent three bicarbonate dialysis sessions per week. The LF-HD group continued dialysis treatments using Lo10–Lo23 dialyzers, whereas patients who were switched from LF-HD to HF-HD changed dialyzers from F6HPS–F10HPS to FX_CorDiax_50 and FX_CorDiax_60.

Fasting blood samples were drawn prior to the dialysis session performed in the middle of the week.

### Laboratory measurements

The same analyst from a single laboratory measured the serum cTnT using a high-sensitivity method (Troponin T hs STAT Elecsys, Roche, Mannheim, Germany). The upper reference limit (99th percentile) was 0.014 ng/ml, and the 95 % CI was 0.0127–0.0249 ng/ml. The blood samples were coded for anonymity prior to laboratory determinations. Other parameters were measured using routine laboratory methods.

### Statistical analysis

Coded data were the subject of statistical analysis.

The results are shown as the means ± SD or median and range, in accordance with the distribution of variables. Qualitative parameters are given as percentages. The differences in continuous variables observed during the prospective study (∆) were calculated as the difference between the results obtained at the analyzed week of the study and those detected at the start of the study (∆ values exceeding 0 indicate an increase during the study, whereas ∆ values below 0 indicate a decrease during the study).

Correlations between continuous variables were determined after calculating the Spearman rank correlation coefficient. The multivariate adaptive regression splines (MARSplines) model with generalized cross-validation (GCV) was used to show indicators of serum cTnT concentration. The receiving operating characteristic (ROC) curve methodology was applied to determine the predictive values of the eQB rate or QB/QD with respect to the serum cTnT concentrations. The results of the continuous variables were compared using the Mann–Whitney test, Student’s *t* test, or the Kruskal–Wallis test, with post hoc analyses, where applicable. The Q-Cochran test or Chi-square test (with or without Yates correction) was applied to compare dichotomous variables, where appropriate. A general linear model (GLM) multiway repeated measures analysis was used to compare the results obtained during the prospective study.

The Kaplan–Meier method, followed by the log-rank test, was used to estimate the significance of the differences in cumulative proportion surviving curves characterizing the tested groups.

A *P* value below 0.05 was considered statistically significant.

The aforementioned statistical calculations were performed using GraphPad InStat 3.10, 32 bit for Windows, created July 9, 2009 (GraphPad Software, Inc., La Jolla, CA, USA), CytelStudio version 10.0, created January 16, 2013 (CytelStudio Software Corporation, Cambridge, MA, USA), and Statistica version 10, 2011 (Stat Soft, Inc., Tulsa, OK, USA).

## Results

### Results of the cross-sectional study

The patients dialyzed using different ED modalities did not differ in serum cTnT concentrations (Table 1). The eQB rate was significantly higher during HDF than during HF-HD or LF-HD (Table 2). The correlations between the cTnT values and the eQB rates among the HDF, HF-HD, or LF-HD groups were not significant.

In the entire ED group, the cTnT was 0.032, 0.008–0.595 ng/ml, the eQB rate was 304 ± 54 ml/min, the QD rate was 499 ± 54 ml/min, and the QB/QD was 62 ± 11 %. In the MARSplines model (GCV 0.001, corr. *R*^2^ = 0.843), the strength of the cTnT indicators in the entire ED group was observed in the following order: RRT vintage (number of references to this factor 19), age (18), eQB rate (17), serum phosphorus (16), serum C-reactive protein (CRP) (14), blood bicarbonate (11), diabetic nephropathy (10), atrial fibrillation (9), QB/QD (3), serum albumin (2), high (arm or proximal forearm) placement of the arteriovenous fistula (2), cerebral stroke (2), poor control of hypertension (2), cardiomyopathies (1), and coronary artery disease (0). Higher cTnT concentrations were primarily associated with longer RRT vintage, older age, and higher eQB rate; lower cTnT was observed concomitantly with higher serum albumin and blood bicarbonate concentrations.

**Fig. 1:**
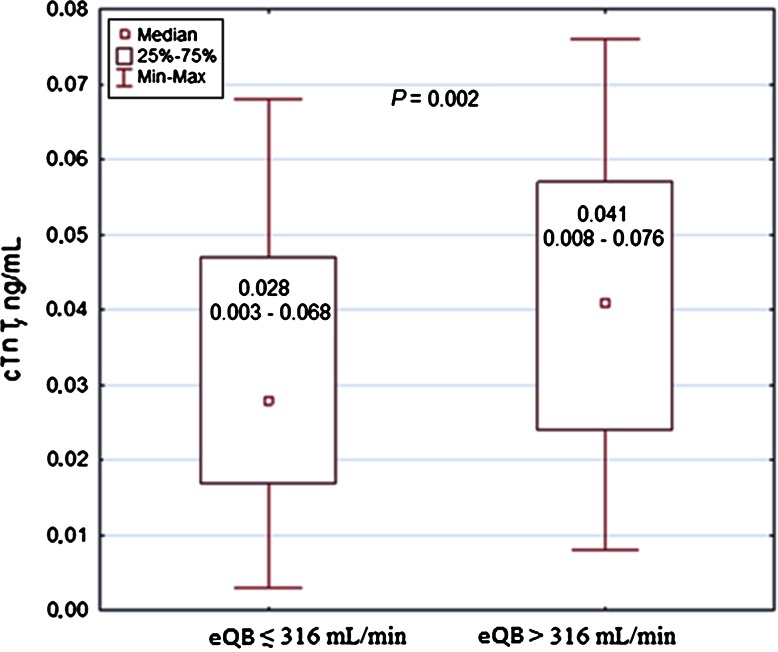
Cardiac troponin T in dialysis patients in relation to the effective blood flow rate. *cTnT* cardiac troponin T, *eQB* the effective blood flow rate. Number of patients equals 186; patients being in the upper cTnT quartile (*n* = 61) are excluded

**Fig. 2:**
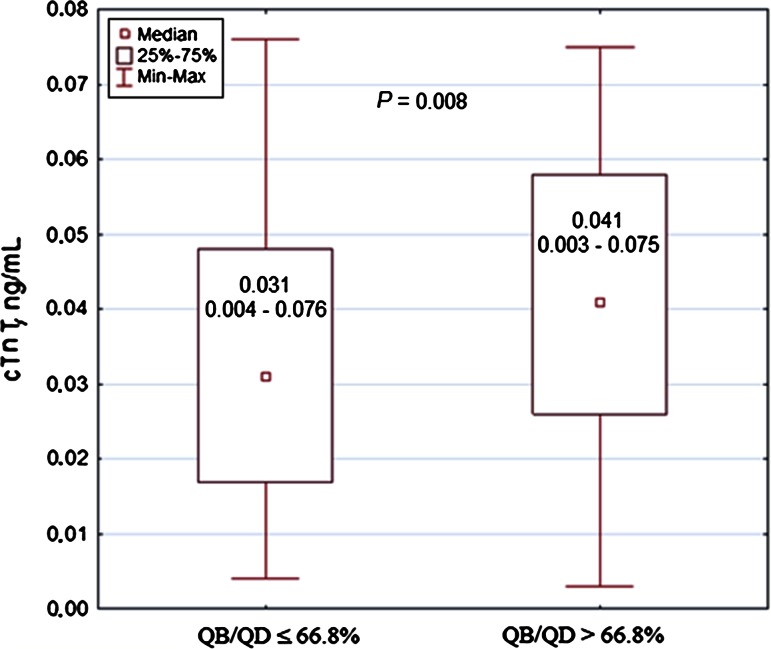
Cardiac troponin T in dialysis patients in relation to the effective blood flow-to-dialysate flow ratio. *cTnT* cardiac troponin T, *QB/QD* the effective blood flow-to-dialysate flow ratio. Number of patients equals 186; patients being in the upper cTnT quartile (*n* = 61) are excluded

In all ED patients, the correlations between the cTnT values and the eQB rate or QB/QD were not significant. When patients were divided according to quartiles of cTnT concentrations (Q1 and Q4 presented the lowest and the highest cTnT values, respectively), a weak positive correlation between cTnT and the eQB rate was observed for the combined values of Q1–Q3 quartiles (*r* = 0.177, *P* = 0.015) (Supplementary Figure 1). An eQB of 316 ml/min and QB/QD of 66.8 % were indicated in the ROC curve methodology as the cutoff values differentiating Q1–Q3 patients with respect to cTnT concentrations. Subjects dialyzed with eQB ≤ 316 ml/min or QB/QD ≤ 66.8 % had significantly lower cTnT compared with patients dialyzed with QB > 316 ml/min or QB/QD > 66.8 % (Figs. 1, 2, respectively). The Q4 patients, with disturbed significance of correlation in the entire ED group, had cTnT more than fivefold higher (>0.076 ng/ml) than the upper reference cTnT 99th percentile limit (0.014 ng/ml), representing seriously ill individuals compared with the remaining subjects (Table 4). Categorical variables associated with the highest cTnT concentrations included atrial fibrillation, diabetes mellitus, congestive heart failure, mitral valvular disease, cardiomyopathies, coronary artery disease, and chronic obstructive pulmonary disease (Table 4, Supplementary Table 1). Among all ED patients, 58 subjects did not exhibit any of the aforementioned comorbidities. Among this group, a positive correlation between cTnT and eQB was revealed (*r* = 0.271, *P* = 0.040). There were no significant correlations between cTnT and eQB in groups showing comorbidities, independent of their number (Table 5, Supplementary Table 1). Notably, a negative correlation was frequently observed between cTnT and eQB in patients with comorbidities. In the group including patients with five comorbid conditions in each case and simultaneously showing the highest median cTnT, the Spearman correlation coefficient of −0.285 had an absolute value similar to that observed in patients without comorbidities, but this value was not significant in a total of ten subjects (Table 5).

**Table 4:** Comparison of demographic, clinical, and laboratory parameters of extracorporeal dialysis patients having serum cardiac troponin T concentration in the upper quartile with the respective data of remaining patients

Parameter	cTnT quartile 4	cTnT quartiles 1–3	*P* value
	*N* = 61	*N* = 186	
Male gender (*n*, %)	40 (65.6)	98 (52.7)	0.08^C^
Age (years)	67.67 (26.3–86.3)	63.7 (23.1–91.8)	0.02^MW^
Diabetes mellitus (*n*, %)	28 (45.9)	44 (23.7)	0.0009^C^
Diabetic nephropathy (*n*, %)	25 (41.0)	36 (19.4)	0.0007^C^
Coronary artery disease (*n*, %)	28 (45.9)	57 (30.7)	0.03^C^
Myocardial infarction (*n*, %)	20 (32.8)	39 (21.0)	0.06^C^
Cardiomyopathies (*n*, %)	26 (42.6)	45 (24.2)	0.006^C^
Mitral valvular disease (*n*, %)	15 (24.6)	17 (9.1)	0.004^Y^
Aortal valvular disease (*n*, %)	2 (3.3)	7 (3.8)	0.8Y
Atrial fibrillation (*n*, %)	13 (21.3)	8 (4.3)	0.0001^Y^
Congestive heart failure	55 (90.2)	111 (59.7)	<0.001^C^
NYHA (class III–IV) (*n*, %)	22 (36.1)	21 (11.3)	0.001^C^
Dialysis modality			
HDF	14 (22.9)	40 (21.5)	0.3^C^
HF-HD	5 (8.2)	30 (16.1)	
LF-HD	42 (68.9)	116 (62.4)	
eQB (ml/min)	307 (158–410)	312 (148–420)	0.347^MW^
Poor control of hypertension (*n*, %)	7 (11.5)	30 (16.2)	0.5^Y^
Cerebral stroke (*n*, %)	8 (13.1)	10 (5.4)	0.08^Y^
COPD (*n*, %)	8 (13.1)	8 (4.3)	0.03^Y^
RRT vintage (years)	2.45 (0.11–25.92)	2.43 (0.019–20.65)	0.5^MW^
Permanent catheter (*n*, %)	15 (24.6)	40 (21.5)	0.6^C^
Albumin (g/l)	40 (26–48)	41 (18–49)	0.1^MW^
cTnT (ng/ml)	0.111 (0.077–0.595)	0.035 (0.003–0.076)	<0.0001^MW^
CRP (mg/l)	8.7 (0.8–241.2)	5.7 (0.4–113.8)	0.04^MW^
β_2_-Microglobulin (mg/dl)	3.9 (0.085–7.72)	2.78 (0.085–13.9)	0.01^MW^
Ca (mg/dl)	8.9 (6.3–11.4)	8.69 (5.1–11)	0.1^MW^
P (mg/dl)	4.5 (1.6–11.1)	4.7 (2.1–10.5)	0.7^MW^
PTH (ng/l)	233 (9.1–1900)	307.5 (4–2002)	0.1^MW^
ALP (IU/l)	98 (42–446)	91 (39–725)	0.1^MW^
Bicarbonate (mmol/l)	21.7 (13.9–28.3)	21.9 (11.8–30.4)	0.5^MW^

Results are shown as median and range or as numbers with percentage

Statistical tests: C—Chi-square, MW—Mann–Whitney, Y—Chi-square with Yates correction

Conversion factors to SI units are as follows: for CRP—9.524, for Ca—0.25, for P—0.323, for ALP—0.0167

*ALP* total alkaline phosphatase, *COPD* chronic obstructive pulmonary disease, *CRP* C-reactive protein, *cTnT* cardiac troponin T, *eQB* effective extracorporeal blood flow, *HDF* hemodiafiltration, *HF-HD* high-flux hemodialysis, *LF-HD* low-flux hemodialysis, *NYHA* New York Heart Association, *PTH* parathyroid hormone

A *P* value of <0.05 is considered statistically significant

**Table 5:** Correlation of serum cTnT concentration and eQB in cross-sectional patients categorized by number of comorbidities (*n* = 247)

Number of comorbidities^a^	Number of patients	cTnT (ng/ml)	eQB (ml/min)	Spearman rank correlation	
				*r*	*P*
0	58	0.025, 0.003–0.170	312, 160–407	0.271	0.040
1	54	0.043, 0.009–0.410	305.5, 156–395	−0.172	0.214
2	56	0.056, 0.010–0.207	301, 160–400	0.060	0.659
3	50	0.052, 0.009–0.595	311.5, 159–398	−0.098	0.497
4	19	0.075, 0.010–0.201	322, 148–420	−0.017	0.946
5	10	0.110, 0.030–0.315	309.5, 189–393	−0.285	0.427
4/5	19/10	0.082, 0.010–0.315	320, 148–420	−0.111	0.565

Results are shown as median and range

*cTnT* cardiac troponin T, *eQB* effective extracorporeal blood flow

^a^Comorbidities: atrial fibrillation, cardiomyopathies, chronic obstructive pulmonary disease, congestive heart failure, coronary artery disease, diabetes mellitus, mitral valvular disease

A *P* value of <0.05 is considered statistically significant

The outcomes for 154 patients, who did not show changes in the ED modality for up to 42 months, were dependent on the initial cTnT concentrations (HR 1.97, 95 % CI 1.39–2.80, *P* = 0.0002 for all-cause mortality; HR 2.18, 95 % CI 1.32–3.60, *P* = 0.002 for cardiac mortality), but were not associated with the initial eQB (*P* = 0.3 for all-cause mortality; *P* = 0.4 for cardiac mortality). The cumulative proportion surviving curves are presented in Supplementary Figures 2–5.

### Results of the prospective observational study

The initial demographic, clinical, and laboratory parameters for patients who finished the prospective study without symptomatic cardiac events are shown in Supplementary Table 2.

**Fig. 3:**
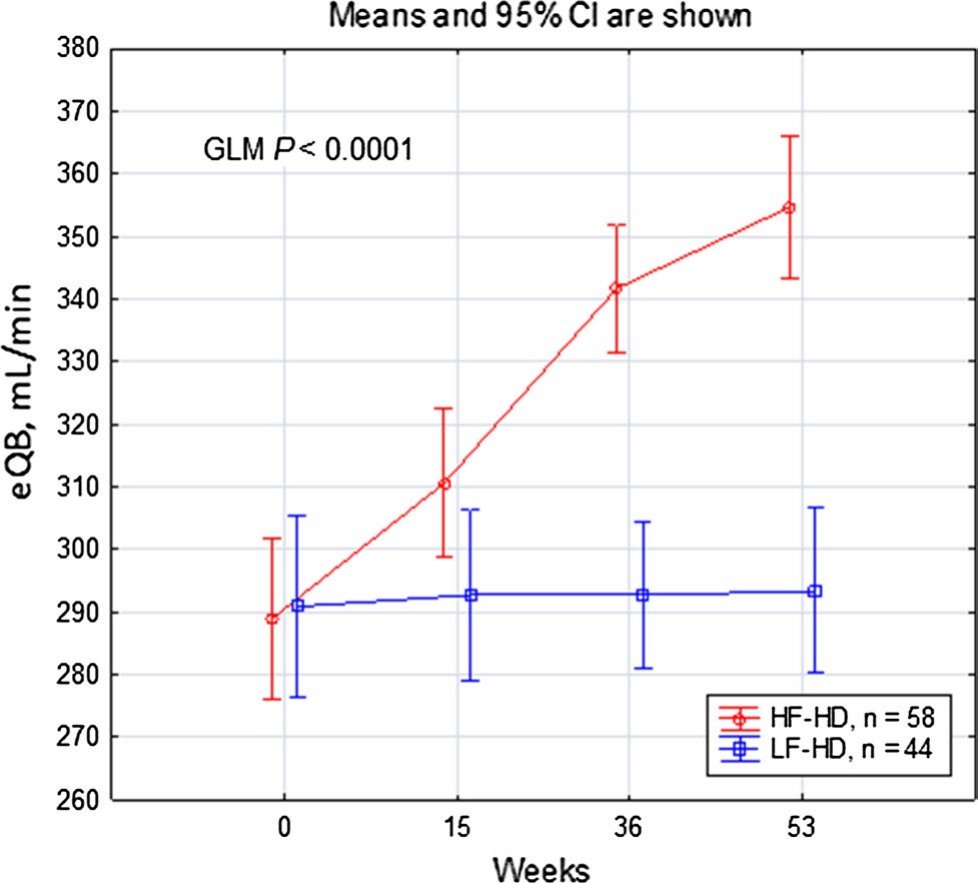
Effective blood flow during a prospective study in patients using high-flux hemodialysis or low-flux hemodialysis. *HF-HD* high-flux hemodialysis, *LF-HD* low-flux hemodialysis, *eQB* the effective blood flow rate

The eQB rate during HF-HD increased from 289 ± 49 to 355 ± 45 ml/min (Fig. 3), whereas the QD rate decreased from 497 ± 26 ml/min to 310 ± 45 ml/min (Supplementary Figure 6); both the eQB rate (291 ± 49 ml/min at the start, 293 ± 43 ml/min at the end) and the QD rate (500 ml/min) were stable during LF-HD. Differences in both parameters were highly significant between and within the HF-HD group (GLM *P* < 0.0001). The QB/QD increased during HF-HD from 59 ± 12 % to 115 ± 17 %, whereas this value remained stable during LF-HD (58 ± 10 vs. 58 ± 9 %, Supplementary Figure 7).

### Analysis of the HF-HD group

**Fig. 4:**
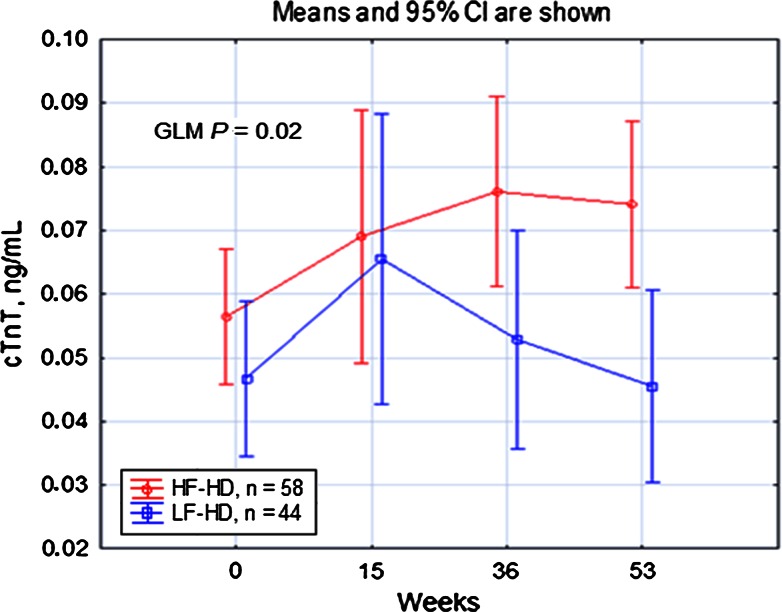
Serum cardiac troponin T concentrations during a prospective study in patients using high-flux hemodialysis or low-flux hemodialysis. *cTnT* cardiac troponin T, *HF-HD* high-flux hemodialysis, *LF-HD* low-flux hemodialysis. Significant differences in the post hoc GLM analysis: HF-HD versus LF-HD: 36th week: *P* = 0.045, 53rd week: *P* = 0.01. HF-HD course: 0 versus 36th week: *P* = 0.004, 0 versus 53rd week: *P* = 0.01; LF-HD course: 0 versus 15th week: *P* = 0.02, 15th versus 53rd week: *P* = 0.01

We also analyzed the cTnT concentrations during the HF-HD course in 58 patients who finished the study without symptomatic cardiac events (Fig. 4), and significantly higher cTnT values were observed at 36 and 53 weeks compared with the start of the study. The MARSplines model (GCV 0.0008, corr. *R*^2^ = 0.872) indicated an increase in the cTnT concentration between the start and end of the study (∆cTnT of 0.007, −0.046 to +0.201 ng/ml), associated with the initial cTnT concentration (15 references), an increase in the eQB (7 references for ∆eQB of 62, 8–226 ml/min), and a decrease in the serum albumin (5 references for ∆albumin of −2, −10 to +10 g/dl). The ∆cTnT was negatively correlated with the initial cTnT concentration (*r* = −0.436, *P* = 0.0006, *n* = 58). Although the entire HF-HD group showed a significant increase in the cTnT concentration during the prospective study, the cTnT increased in 36 patients (∆cTnT 0.022, 0.001–0.201 ng/ml), whereas in 22 patients, either no difference or a decrease in cTnT (∆cTnT −0.015, 0.000 to −0.046 ng/ml) was observed. Patients showing increased cTnT had lower initial cTnT compared with those showing decreased cTnT (0.035, 0.009–0.268 vs. 0.063, 0.026–0.141, *P* = 0.002). The initial and increased eQB values were similar in both groups (*P* = 0.250 and *P* = 0.374, respectively) during the study.

Other changes in clinical/laboratory parameters were not significant or indicated improvement compared with the beginning of the study. The latter was observed in the case of the decreased administration of anti-hypertensive medication (Supplementary Figure 8), an increase in eKt/V (1.29, 0.54–1.69 vs. 1.38, 0.70–1.64, *P* < 0.05) and HDL-cholesterol (40, 21–90 vs. 44.5, 24–86 mg/dl, *P* = 0.011), and a decrease in β_2_-microglobulin (3.8, 0.99–13.9 vs. 2.6, 0.73–5.25 mg/dl, *P* < 0.05).

### Analysis of the LF-HD group

During LF-HD course, an increase in cTnT in the 44 patients who finished the study without symptomatic cardiac events was observed between the start and the 15th week of the study (Fig. 4). The MARSplines model (GCV 0.00006, corr. *R*^2^ = 0.999) showed that this increase in cTnT concentration was influenced by an increase in serum phosphorus (21 references for ∆phosphorus of 0.44, −1.80 to +3.52 mg/dl), older age (10 references), and diabetic nephropathy (9 references). The serum phosphorus levels observed after 15 weeks were higher in 31 (70.5 %) of the 44 LF-HD patients compared with the initial phosphorus values. Thus, an intense decrease in serum phosphorus through dietary changes and the administration of pharmacological medication became the therapeutic goal in all LF-HD subjects showing serum phosphorus levels higher than the reference limit. In response, the cTnT concentration increased, while the phosphorus concentration decreased, and at the end of the study, the levels of these parameters were not different from those observed at the beginning of the study (Fig. 4, Supplementary Figure 9).

LF-HD patients with stable eQB during the 53 weeks of the study were divided into two subgroups according to the initial eQB. These subgroups were analyzed separately and compared to determine whether differences in the initial eQB affected the final concentrations of plasma cTnT. Among this group, 22 patients were dialyzed with eQB < 300 ml/min, and the median initial cTnT was 0.039 (0.011–0.127) ng/ml, while the remaining 22 subjects were dialyzed with eQB ≥ 300 ml/min, and the initial cTnT was 0.045 (0.004–0.160) ng/ml. After 53 weeks of prospective observation with unchanged eQB, the cTnT levels were similar (0.039, 0.015–0.134 ng/ml and 0.045, 0.006–0.122 ng/ml, respectively).

During the 53 weeks of the prospective study, the serum albumin level (41.5 ± 3.5 vs. 34.3 ± 3.9 g/dl, *P* < 0.0001) and HDL-cholesterol (40, 23–73 vs. 37, 21–71 g/dl, *P* < 0.05) were decreased, whereas the β_2_-microglobulin (2.35, 0.09–6.28 vs. 3.51, 0.50–9.97 mg/dl, *P* < 0.05), parathyroid hormone (PTH, 481, 4–1900 vs. 600, 5–1900 pg/ml, *P* < 0.05), and total alkaline phosphatase (ALP, 95, 47–354 vs. 123, 78–687 U/l, *P* < 0.05) were increased in LF-HD patients.

### Comparison of the HF-HD and LF-HD groups

Patients of both prospective groups initially showed similar cTnT at the beginning of the study (Fig. 4). During the study, significant differences in serum cTnT concentrations were revealed between both HD modalities (GLM *P* = 0.019): Compared with the LF-HD group, higher cTnT levels were observed in the HF-HD group at 36 and 53 weeks. Additionally, both groups differed in serum CRP, with a decrease observed during HF-HD and an increase observed during LF-HD (GLM *P* < 0.008). A similar pattern of changes was observed for β_2_-microglobulin (GLM *P* < 0.0001). The time-dependent increments for ALP (GLM *P* = 0.0005) and PTH (GLM *P* < 0.0001) were more pronounced in LF-HD than in HF-HD patients. GLM analysis indicated higher serum phosphorus during LF-HD than during HF-HD (GLM *P* < 0.0006), but this difference was ameliorated by dietary and pharmacological treatments in the LF-HD patients. A decrease in serum albumin was observed in both groups, but this decrease was more evident in LF-HD patients (GLM *P* < 0.0001). The control of hypertension improved during HF-HD, but remained worse in LF-HD patients (Supplementary Figure 8).

During the prospective study, there were no significant differences between the all-cause and cardiac mortalities and frequency of non-fatal cardiac episodes in both groups (Table 3). At 21 months after the study was completed, LF-HD and HF-HD patients did not differ in all-cause and cardiac mortality rates (Supplementary Figures 10 and 11), although these patients significantly differed in eQB (293 ± 43 vs. 355 ± 45 ml/min, respectively, *P* < 0.000001) and cTnT (0.046 ± 0.031 vs. 0.074 ± 0.061 ng/ml, respectively, *P* = 0.01) at the beginning of the 21-month observation period.

## Discussion

No correlation between the QB rate and cardiac troponin levels was observed in previous studies [12, 13], although one study showed a positive weak correlation [4]. There was also no such correlation observed in the present study after analyzing all ED patients included in the cross-sectional study.

The lack of a direct correlation between eQB and cTnT might reflect multiple factors influencing cardiac troponins [4, 6, 8, 9, 11], which obviously differentiate ED patients, independent from the eQB rate. Nevertheless, a predictive value for the eQB rate with respect to the serum cTnT was detected in the present study for the ED group among other well-known cTnT determinants, including dialysis vintage [4, 5], age [8], diabetes [6], serum phosphate [9], or serum CRP [4–6]. A significant correlation between eQB and cTnT was observed at lower cTnT concentrations, particularly in patients without any comorbidities (median cTnT of 0.025 ng/ml) or patients with less advanced comorbid conditions and cTnT levels in the Q1–Q3 quartiles (median cTnT of 0.035 ng/ml). In patients with high cTnT resulting from multiple contributing factors, the serum cTnT did not correlate with the eQB; indeed, this correlation was rather negative, suggesting a lower cTnT at the higher QB rates. It is reasonable to speculate that the response to the eQB rate might be different in ED patients without comorbid conditions, in whom higher eQB rates contribute to myocardial injury and increased serum cTnT, and in ED patients suffering from multiple comorbidities, primarily of cardiac origin, higher eQB might decrease cTnT.

In the course of ED therapy, serum cTnT levels might increase, reflecting influences of uremia and dialysis-associated factors on myocardium [2, 3, 5, 8, 12–15]. Based on patients treated with LF-HD without any changes in eQB and QD, these factors were considered as indicators of time-dependent differences in serum cTnT levels. At the start of the prospective study, compared with HF-HD patients, LF-HD subjects were younger and had better control of hypertension, no cerebral strokes in the past, lower β_2_-microglobulin levels, and higher PTH concentrations. However, despite these differences, initial serum cTnT concentrations were comparable in both groups.

The prospective study revealed that the gradual increase in eQB at an average rate of 66 ml/min did not result in significant differences in the frequencies of both cardiovascular mortality and non-fatal cardiac episodes compared with the respective frequencies for patients dialyzed with stable eQB. To determine the impact of increasing eQB rates on serum cTnT concentrations as an indicator of silent myocardial injury, patients showing symptomatic cardiac episodes and instability of non-cardiac origin were not included in the analyses, and the results revealed that the serum cTnT concentration might increase during HD treatment, even in stable patients, free from symptomatic cardiac events and without other remarkable changes in health status. Increases in serum cTnT were associated with higher cTnT concentrations in the present cross-sectional study (age, eQB, serum phosphorus, serum CRP, diabetic nephropathy, and serum albumin), consistent with previous studies [4–7, 9]. The factors likely contributing to the increase in cTnT were, however, different in both HD groups showing similar initial cTnT concentrations. These factors did not include those reported as different between the groups at the study beginning. Therefore, the initial differentiating factors did not affect changes in cTnT and were not indicated in the well-fitted MARSplines model.

In LF-HD patients, an increase in cTnT was revealed after 15 weeks, and this effect was transient, as the primary contributor, serum phosphorus, was efficiently decreased in the following weeks. In HF-HD patients, elevations in cTnT were evidently associated with increased eQB and QB/QD. Because eQB and QD did not return to the initial values, we cannot determine whether this increase in cTnT was reversible.

In stable HD patients, the serum cTnT increased with gradually increasing eQB. In HD patients using hollow cuprophan fiber dialyzers, an increase in the QB rate from 200 to 400 ml/min with a stable QD rate of 500 ml/min was associated with increased amounts of C3a, the terminal complement complex involving the sequential assembly of the precursor proteins C5b and C6–C9 [16]. Additional biocompatible dialysis membranes generated lower but detectable increments in C3a [17, 18]. In experimental studies evaluating the role of C5 and C3a in myocardial ischemia, Busche et al. [19] showed that C3a and C5 were involved in increments of serum cTnI concentrations following myocardial ischemia/reperfusion injury. Therefore, myocardial ischemia/reperfusion injury might represent a potential mechanism of myocardial injury in patients with higher QB rates.

However, the results of the present study also revealed that increased cTnT was negatively correlated with the initial cTnT concentration when an increase in the eQB was observed during HF-HD. Patients with high cTnT concentrations resulting from multiple contributing factors might show no or lower increments or even a decrease in cTnT levels when eQB is high during HF-HD. This observation is consistent with the results of the present cross-sectional study.

However, the factors causing the increase or decrease in cTnT in patients with higher initial cTnT levels during the prospective study remain unknown, and whether a decrease in serum cTnT concentration reflects a decrease in myocardial injury or only a removal of cTnT from the blood as a result of the use of more efficient HD compared with previously applied therapies remains to be determined.

HF-HD diminishes uremic toxicity more effectively than LF-HD [20–22], suggesting a beneficial effect on myocardium, although HF-HD is associated with higher eQB rates. The benefits of HF-HD compared with LF-HD were revealed in the present study with respect to serum albumin, CRP, β_2_-microglobulin, PTH, ALP, and phosphorus. Uremic patients showing worsened conditions (diabetic subjects) or biochemistries (serum albumin ≤ 4 g/dl) showed improved survival on HF-HD compared with LF-HD [20]. The benefits of HF-HD might at least partially counterbalance the negative effects of increases in both eQB and QB/QD and elevations in serum cTnT. According to Palmer et al. [23], cardiovascular mortality was reduced by 17 % in patients receiving HF-HD. However, these authors did not analyze eQB.

However, when high-efficiency dialysis techniques requiring higher eQB rates are applied to patients with high cTnT concentrations reflecting comorbid diseases, the serum cTnT, similar to other proteins, might decrease during transmembrane leakage from the blood to the dialysate. This phenomenon is particularly relevant for proteins with lower molecular weights [24, 25]. In patients receiving HF-HD in the prospective study, a significant protein decrease was observed for β_2_-microglobulin. However, in a recent study, Laveborn et al. [26] indicated that HF-HD or HDF might mask increases in the cTnT concentration, reflecting the removal of cTnT and associated fragments during ED using more efficient dialysis techniques. A decrease in natural cTnT concentrations disrupts the relationship between cTnT and tested factors, such as eQB or survival. Thus, it is likely that the evaluation of echocardiographic parameters closely associated with serum cTnT (E/E`, right ventricular Tei index, left ventricular ejection fraction [27]) might be helpful in determining whether a decrease in cTnT levels indicates the amelioration of cardiac damage during the transmembrane loss of cTnT.

Serum cTnT is an independent predictor of all-cause and cardiovascular mortalities in HD patients [28], as confirmed in the present study. The eQB rate was not associated with all-cause or cardiac mortality in ED patients, the cross-sectional group or the prospective groups.

A lack of association between eQB and survival might reflect the following conditions:

- A direct relationship between eQB and cTnT only at lower serum cTnT concentrations,
- An unclear influence of eQB on serum cTnT in asymptomatic patients with high cTnT concentrations,
- The removal of cTnT to the dialysate during HF-HD or HDF,
- The amelioration of the potential negative cardiac effects of higher eQB rates, and
- The analysis of survival rates based on the initial eQB rates, considering that the eQB might differ in consecutive months or years.

Lipschultz et al. [29] suggested that increased serum cTnT concentrations “are not artifactual, but are genuine indicators of cardiomyocyte damage.” Therefore, elevations in cTnT without cardiac symptoms should be seriously considered and prevented. The results of the present study indicated that a decrease in the elevated serum phosphorus concentration, associated with increased cTnT, might restore cTnT concentrations to the initial levels. When patients are maintained under relatively stable conditions during HD, the serum cTnT levels might also remain unchanged at least up to 53 weeks, irrespective of the eQB rates, consistent with the prospective observations in LF-HD patients.

In summary, in patients receiving stable ED, silent ongoing myocyte injury, indicated as increased serum cTnT concentrations, is primarily associated with comorbidities, longer RRT vintage, older age, and high serum phosphorus and CRP concentrations. Higher eQB rates increase cTnT levels, thereby resulting in myocardial damage during ED in subjects with lower cTnT concentrations, which does not affect the outcome of ED patients. The impact of higher eQB rates on further myocardial injury in patients showing high cTnT levels remains unclear and requires additional studies.

## Electronic supplementary material

Supplementary material 1 (DOC 549 kb)
