# Supplementary material for: Serum cardiac troponin T and effective blood flow in stable extracorporeal dialysis patients
Source: Int Urol Nephrol. 2015 Nov 24;48:419–29. doi: 10.1007/s11255-015-1165-z (PMC4769722; doi:10.1007/s11255-015-1165-z)
Supplement: Supplementary file 1 — Supplementary material 1 (DOC 549 kb) [file 11255_2015_1165_MOESM1_ESM.doc]

***Supplementary Material***

***Supplementary*** ***Table 1***. Correlation of serum cTnT concentration and eQB in cross-sectional patients categorized according to kinds of comorbidity (n = 189)

| Comorbidity | Number of patients | cTnT  ng/ ml | eQB  ml/min | Spearman rank correlation  r *P* |
| --- | --- | --- | --- | --- |
| Atrial fibrillation | 21 | 0.092, 0.011-0.261 | 312, 247-393 | -0.040 0.864 |
| COPD | 16 | 0.082, 0.010-0.315 | 316, 148-393 | -0.064 0.814 |
| Mitral valvular disease | 32 | 0.069, 0.009-0.230 | 314, 180-393 | -0.234 0.198 |
| Cardiomyopathies | 71 | 0.064, 0.009-0.315 | 310, 148-420 | 0.026 0.830 |
| Diabetes mellitus | 72 | 0.064, 0.010-0.595 | 310, 148-420 | -0.019 0.874 |
| Coronary artery disease | 85 | 0.058, 0.009-0.595 | 320, 158-420 | -0.031 0.777 |
| Congestive heart failure | 166 | 0.057, 0.009-0.595 | 307.5, 148-420 | 0.053 0.498 |

Results are shown as median and range.

A *P* value of <0.05 is considered statistically significant.

Abbreviations: COPD – chronic obstructive pulmonary disease, cTnT - cardiac troponin T, eQB – effective extracorporeal blood flow

Majority of patients (n = 135) showed more than one comorbidity.

***Supplementary*** ***Table 2***. Initial demographic, clinical and laboratory parameters of patients who finished the prospective study without symptomatic cardiac events

| Parameter | LF-HD  n = 44 | HF-HD  n = 58 | *P* value |
| --- | --- | --- | --- |
| Male gender, n, % | 20 (45.5) | 28 (48.3) | 0.8^C^ |
| Age, years | 62.1 ± 14 | 67.7 ± 12.7 | 0.04^S^ |
| Diabetes, n, % | 8 (18.2) | 17 (29.3) | 0.2^C^ |
| Diabetic nephropathy, n, % | 8 (18.2) | 11 (19.0) | 0.9^C^ |
| Chronic glomerulonephritis, n, % | 15 (34.1) | 12 (20.7) | 0.1^C^ |
| Hypertensive nephropathy, n, % | 6 (13.6) | 8 (13.8) | 0.8^Y^ |
| Chronic tubulointerstitial nephritis, n, % | 0 (0.0) | 6 (10.3) | 0.04^F^ |
| Coronary artery disease, n, %   - myocardial infarction, n, % | 5 (11.4)  3 (6.8) | 15 (25.9)  10 (17.2) | 0.1^Y^  0.2^Y^ |
| Cardiomyopathies, n, % | 9 (20.5) | 18 (31.0) | 0.2^C^ |
| Mitral valvular disease, n, %  Aortal valvular disease, n, % | 9 (20.5)  0 (0.0) | 8 (13.8)  3 (5.2) | 0.5^Y^  0.3^F^ |
| Atrial fibrilation, n, % | 3 (6.8) | 8 (13.8) | 0.8^Y^ |
| Cerebral stroke, n, % | 0 (0.0) | 13 (22.4) | 0.0005^F^ |
| Poor control of hypertension, n, % | 0 (0.0) | 13 (22.4) | 0.0005^F^ |
| COPD, n, % | 1 (2.3) | 5 (8.6) | 0.4^Y^ |
| RRT vintage, years | 3.5 (0.07-19.1) | 2.1 (0.37-15.4) | 0.3^MW^ |
| BMI, kg/m^2^ | 27.9 (19.7-45.1) | 26.5 (18.9-55.3) | 0.3^MW^ |
| Albumin, g/l | 42 (30-49) | 42 (25-48) | 1.0^MW^ |
| CRP, mg/l | 7.6 (1.0-113.8) | 8.1 (1.0-161.5) | 0.8^MW^ |
| cTnT, ng/ml | 0.042  ( 0.004-0.160) | 0.047  (0.009-0.268) | 0.4^MW^ |
| β_2_-microglobulin, mg/dl | 2.6 (0.09-6.3) | 3.8 (0.99-13.9) | 0.003^MW^ |
| Ca, mg/dl | 8.7 (7.2-10.6) | 8.9 (7.8-11.4) | 0.2^MW^ |
| P, mg/dl | 4.7 (2.4-9.7) | 4.7 (2.5-10.5) | 0.9^MW^ |
| PTH, ng/l | 475.5 (4-1900) | 192.7 (40.3-1401) | 0.008^MW^ |
| ALP, IU/l | 94.5 (47-354) | 99 (39-487) | 0.9^MW^ |
| Blood Ph | 7.4 ± 0.04 | 7.4 ± 0.04 | 0.06^S^ |
| Bicarbonate, mmol/l | 21.6 (17.9-23.9) | 21.9 (15.4-26.3) | 0.1^MW^ |

Statistical tests: C – Chi square, F – Fisher exact, MW – Mann Whitney, S – t-Student, Y – Chi square with Yates correction

Abbreviations: ALP – total alkaline phosphatase, BMI - body mass index, COPD – chronic obstructive pulmonary disease, CRP – C-reactive protein, cTnT - cardiac troponin T, HF-HD – high-flux hemodialysis, LF-HD – low-flux hemodialysis, PTH – parathyroid hormone, RRT – renal replacement therapy vintage

Results are shown as median and range, mean ± standard deviation or as numbers with percentage.

A *P* value of <0.05 is considered statistically significant.

Conversion factors to SI units are as follows: for CRP – 9.524, for Ca - 0.25, for P - 0.323, for ALP - 0.0167

***Supplementary Figure 1.*** Real blood flow in dialysis patients separated using quartile levels of serum cardiac troponin T

cTnT - cardiac troponin T; eQB – the effective blood flow rate

***
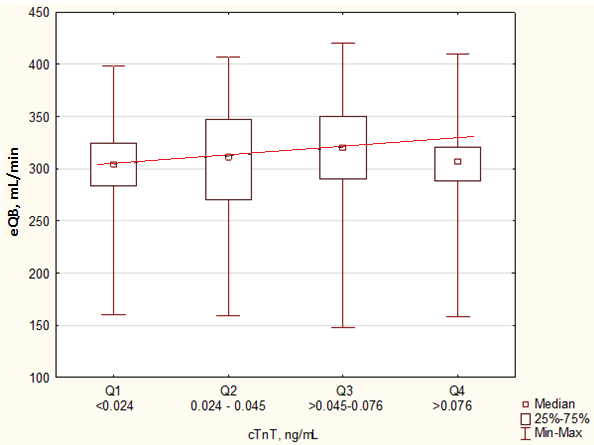
***

Q1 – Q3 cTnT and eQB: *r* = 0.177, *P* = 0.015

eQB of Q1 vs Q3 *P* = 0.032

eQB of Q3 vs Q4 *P* = 0.041

**When the eQB rate was checked in cTnT quartiles, it could be shown that eQB r**esults of patients being in the upper cTnT quartile “disturbed” a positive correlation between cTnT and the eQB rate.

***Supplementary Figure 2.*** A**ll-cause mortality in 154 patients who did not change dialysis modality up to 42 months in relation to tertiles (T) of the initial** cardiac troponin T **(cTnT) concentrations**

Multiple-sample test *P* = 0.0002

T_1_ = cTnT ≤ 0.029 ng/mL

T_2_ = cTnT (0.029, 0.062> ng/mL

T_3_ = cTnT > 0.062 ng/mL

T_1_ vs T_2_: HR 2.96, 95% CI 1.31 - 6.69, *P* = 0.009

T_1_ vs T_3_: HR 2.07, 95% CI 1.39 - 3.09, *P* = 0.003

T_1_ vs T_2_ vs T_3_: HR 1.97, 95% CI 1.39 - 2.80, *P* = 0.0002

***Supplementary Figure 3.*** C**ardiac mortality in 127 patients who did not change dialysis modality up to 42 months in relation to tertiles (T) of the initial** cardiac troponin T **(cTnT) concentrations**

Multiple-sample test *P* = 0.005

T_1_ = cTnT ≤ 0.029 ng/mL

T_2_ = cTnT (0.029, 0.062> ng/mL

T_3_ = cTnT > 0.062 ng/mL

T_1_ vs T_3_: HR 2.25, 95% CI 1.29 - 3.93, *P* = 0.004

T_1_ vs T_2_ vs T_3_: HR 2.18, 95% CI 1.32 - 3.60, *P* = 0.002

***Supplementary Figure 4.*** A**ll-cause mortality in 154 patients who did not change dialysis modality up to 42 months in relation to tertiles of the initial eQB**

eQB – the effective blood flow rate

Multiple-sample test *P* = 0.328

***Supplementary Figure 5.*** C**ardiac mortality in 127 patients who did not change dialysis modality up to 42 months in relation to tertiles of the initial eQB**

eQB – the effective blood flow rate

Multiple-sample test *P* = 0.442

***Supplementary Figure 6.*** The dialysate flow rate during a prospective study in patients using high-flux hemodialysis or low-flux hemodialysis

HF-HD - high-flux hemodialysis; LF-HD - low-flux hemodialysis; QD - the dialysate flow rate

***
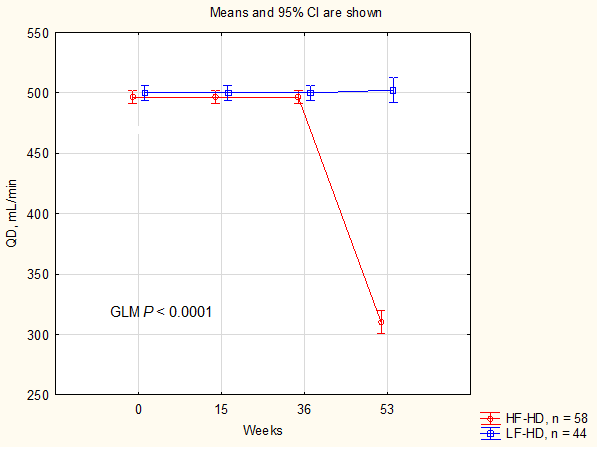
***

***Supplementary Figure 7.*** The effective blood flow to dialysate flow ratio during a 53 week prospective study in patients using high-flux hemodialysis or low-flux hemodialysis

HF-HD - high-flux hemodialysis; LF-HD - low-flux hemodialysis; QB/QD - the effective blood flow to dialysate flow ratio

Significant differences in the post hoc GLM analysis:

HF-HD vs LF-HD

36^th^ week: *P* < 0.0001

53^rd^ week: *P* < 0.0001

HF-HD course:

0 vs 15^th^ week: *P* = 0.003

0 vs 36^th^ week: *P* < 0.0001

0 vs 53^rd^ week: *P* < 0.0001

***
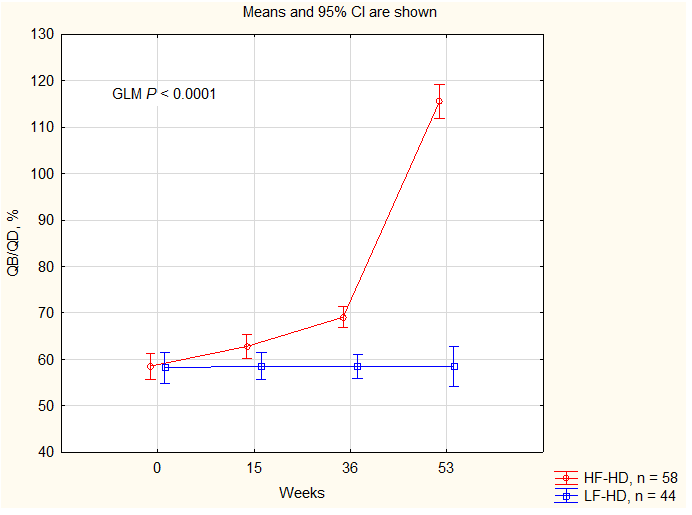
***

***Supplementary Figure 8.*** Administration of anti-hypertensive medication during a prospective study in patients using high-flux hemodialysis or low-flux hemodialysis

HF-HD - high-flux hemodialysis; LF-HD - low-flux hemodialysis


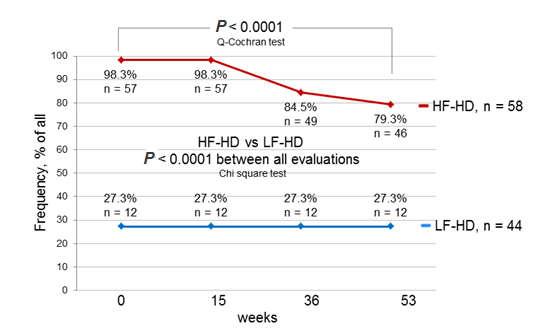


***Supplementary Figure 9.*** Changes in serum phosphorus concentration during a prospective study in patients using low-flux hemodialysis

Friedman test *P* = 0.001

Post hoc *P* < 0.05 for difference between 15th and 53rd study week

***Supplementary Figure 10.*** All-cause mortality rates during 21 months since the end of the study in LF-HD and HF-HD patients

HF-HD – high-flux hemodialysis; LF-HD – low-flux hemodialysis

Test log-rank *P* = 0.166

***Supplementary Figure 11.*** Cardiac mortality rates during 21 months since the end of the study in LF-HD and HF-HD patients

HF-HD – high-flux hemodialysis; LF-HD – low-flux hemodialysis

Test log-rank *P* = 0.318
